# Supplementary material for: Investigation of the neural correlation with task performance and its effect on cognitive load level classification
Source: PLoS One. 2023 Dec 21;18(12):e0291576. doi: 10.1371/journal.pone.0291576 (PMC10735190; doi:10.1371/journal.pone.0291576)
Supplement: S1 Table — (PDF) [file pone.0291576.s001.pdf]

Supplementary Materials

Table S1: Classification accuracy of Different Polynomial Orders in SVM (Numerical Data of Figure 6)

Polynomial Order	Classification Accuracy %
2	73.61
3	55.55
4	93.05
5	77.77
6	50
7	50
