# Supplementary material for: Investigation of the neural correlation with task performance and its effect on cognitive load level classification
Source: PLoS One. 2023 Dec 21;18(12):e0291576. doi: 10.1371/journal.pone.0291576 (PMC10735190; doi:10.1371/journal.pone.0291576)
Supplement: S2 Table — (PDF) [file pone.0291576.s002.pdf]

Supplementary Materials

Table S2: Normalized Channel Power for the Bad Performers (Numerical Data of Figure 7)

Channel No	Rest	Task
1	0.044974	0.080147
2	0.051071	0.075612
3	0.047337	0.06498
4	0.050897	0.074757
5	0.052917	0.070759
6	0.059649	0.067069
7	0.044058	0.064035
8	0.044815	0.0779
9	0.047708	0.072382
10	0.047601	0.064768
11	0.047267	0.078494
12	0.049621	0.058304
13	0.048238	0.075906
14	0.055463	0.057735
15	0.04741	0.064601
16	0.050504	0.079784
17	0.047024	0.076112
18	0.047689	0.073703
19	0.051883	0.065006
