# Supplementary material for: Investigation of the neural correlation with task performance and its effect on cognitive load level classification
Source: PLoS One. 2023 Dec 21;18(12):e0291576. doi: 10.1371/journal.pone.0291576 (PMC10735190; doi:10.1371/journal.pone.0291576)
Supplement: S3 Table — (PDF) [file pone.0291576.s003.pdf]

Supplementary Materials

Table S3: Normalized Channel Power for the Average Performers (Numerical Data of Figure 8)

Channel No	Rest	Task
1	0.058046	0.08185
2	0.076478	0.083944
3	0.069351	0.083999
4	0.063533	0.06558
5	0.043857	0.065558
6	0.050291	0.081612
7	0.079505	0.073549
8	0.08091	0.074413
9	0.07249	0.073139
10	0.059828	0.068777
11	0.062405	0.080262
12	0.057932	0.066961
13	0.06781	0.06616
14	0.054692	0.072688
15	0.075833	0.082817
16	0.059872	0.069816
17	0.065188	0.070665
18	0.057514	0.066765
19	0.071483	0.072298
