# Supplementary material for: Investigation of the neural correlation with task performance and its effect on cognitive load level classification
Source: PLoS One. 2023 Dec 21;18(12):e0291576. doi: 10.1371/journal.pone.0291576 (PMC10735190; doi:10.1371/journal.pone.0291576)
Supplement: S4 Table — (PDF) [file pone.0291576.s004.pdf]

Supplementary Materials

Table S4: Normalized Channel Power for the GOOD Performers (Numerical Data of Figure 9)

Channel No	Rest	Task
1	0.055354	0.070238
2	0.056504	0.071186
3	0.056506	0.063227
4	0.057612	0.069462
5	0.051472	0.056806
6	0.054096	0.068734
7	0.050159	0.063575
8	0.054792	0.062102
9	0.056937	0.063919
10	0.053562	0.067419
11	0.062303	0.057184
12	0.050982	0.06795
13	0.060346	0.066649
14	0.063071	0.067635
15	0.059106	0.070197
16	0.053633	0.065759
17	0.054534	0.066635
18	0.052968	0.061994
19	0.051526	0.064522
