# Supplementary material for: Investigation of the neural correlation with task performance and its effect on cognitive load level classification
Source: PLoS One. 2023 Dec 21;18(12):e0291576. doi: 10.1371/journal.pone.0291576 (PMC10735190; doi:10.1371/journal.pone.0291576)
Supplement: Attachment — Submitted filename: Response to Reviewers.docx [file pone.0291576.s006.docx]

**Response to Reviewers**

Ref	: PONE-D-23-03551R1

Article Title	: Investigation of the neural correlation with task performance and its	effect on cognitive load level classification

Authors	: Farzana Khanam, Mohiuddin Ahmad, and A.B.M. Aowlad Hossain

**Dear Editor in Chief**

**Plos One**

We are thankful for your response to our article and guidelines to improve its quality. Since the reviewers claimed that their queries are addressed properly, this rebuttal letter we prepared to defend the editor’s comment which is:

**Editor Comments:**

“The manuscript has addressed the reviewers' comments and feedback. However it is emphasized that figures (pictures) submitted with the manuscript are not of proper quality and resolution. Please improve the Picture quality and resubmit in the final version.”

***Response to Editor’s Comments:***

Thanks for letting us know that our response to reviewers' comments and feedback was addressed appropriately. We are happy that the reviewers as well as the Editor found potential in this study however the only issue is picture quality.

Due to the concern of image quality, we revised the image quality and improved them accordingly. We hope the revised edition of the images will fulfill the required quality.

Besides, both the previous corrections in the manuscript with track changes and unmarked version of our revised paper without tracked changes are uploaded accordingly.

Thank you again for your effort and contribution to improve our manuscript. We are happy to provide more information to publish this article.

**Sincere Regards,**

**Authors**
